# Post-Irradiation Treatment with a Superoxide Dismutase Mimic, MnTnHex-2-PyP^5+^, Mitigates Radiation Injury in the Lungs of Non-Human Primates after Whole-Thorax Exposure to Ionizing Radiation

**DOI:** 10.3390/antiox7030040

**Published:** 2018-03-07

**Authors:** John Mark Cline, Greg Dugan, John Daniel Bourland, Donna L. Perry, Joel D. Stitzel, Ashley A. Weaver, Chen Jiang, Artak Tovmasyan, Kouros Owzar, Ivan Spasojevic, Ines Batinic-Haberle, Zeljko Vujaskovic

**Affiliations:** 1 Department of Pathology, Section on Comparative Medicine, Wake Forest University School of Medicine, Medical Center Boulevard, Winston-Salem, NC 27157-1040, USA; gdugan@wakehealth.edu (G.D.); perrydl@niaid.nih.gov (D.L.P.); 2 Department of Radiation Oncology, Wake Forest University School of Medicine, Medical Center Boulevard, Winston-Salem, NC 27157-1040, USA; bourland@wakehealth.edu; 3 Integrated Research Facility, Division of Clinical Research, National Institute of Allergy and Infectious Disease, National Institutes of Health, Frederick, MD 21702, USA; 4 Department of Biomedical Engineering, Wake Forest University School of Medicine, Biotech Place, 575 N. Patterson Ave., Winston-Salem, NC 21701, USA; jstitzel@wakehealth.edu (J.D.S.); asweaver@wakehealth.edu (A.A.W.); 5 Department of Biostatistics and Bioinformatics, Duke University Medical Center, Durham, NC 27708, USA; chen.jiang@duke.edu (C.J.); kouros.owzar@duke.edu (K.O.); 6 Department of Radiation Oncology, Duke University Medical Center, Durham, NC 27708, USA; artak.tovmasyan@duke.edu (A.T.); ibatinic@duke.edu (I.B.-H.); zvujaskovic@som.umaryland.edu (Z.V.); 7 Department of Medicine Duke University Medical Center, Durham, NC 27708, USA; ivan.spasojevic@duke.edu; 8 Duke Cancer Institute, Pharmaceutical Research Shared Resource, PK/PD Core Laboratory, Duke University Medical Center, Durham, NC 27708, USA; 9 Department of Radiation Oncology, University of Maryland School of Medicine, 655 W. Baltimore Street, Baltimore, MD 21201, USA

**Keywords:** superoxide dismutase mimetic, MnTnHex-2-PyP^5+^, irradiation, Macaca mulatta, lung injury

## Abstract

Radiation injury to the lung is the result of acute and chronic free radical formation, and there are currently few effective means of mitigating such injury. Studies in rodents indicate that superoxide dismutase mimetics may be effective in this regard; however, studies in humans or large animals are lacking. We hypothesized that post-exposure treatment with the lipophilic mitochondrial superoxide dismutase mimetic, MnTnHex-2-PyP^5+^ (hexyl), would reduce radiation-induced pneumonitis and fibrosis in the lungs of nonhuman primates. Rhesus monkeys (*Macaca mulatta*) received 10 Gy whole thorax irradiation, 10 Gy + hexyl treatment, sham irradiation, or sham irradiation + hexyl. Hexyl was given twice daily, subcutaneously, at 0.05 mg/kg, for 2 months. Animals were monitored daily, and respiratory rates, pulse oximetry, hematology and serum chemistry panels were performed weekly. Computed tomography scans were performed at 0, 2, and 4 months after irradiation. Supportive fluid therapy, corticosteroids, analgesics, and antibiotics were given as needed. All animals were humanely euthanized 4.5 months after irradiation, and pathologic assessments were made. Multifocal, progressive lung lesions were seen at 2 and 4 months in both irradiated groups. Hexyl treatment delayed the onset of radiation-induced lung lesions, reduced elevations of respiratory rate, and reduced pathologic increases in lung weight. No adverse effects of hexyl treatment were found. These results demonstrate (1) development of a nonhuman primate model of radiation-induced lung injury, (2) a significant mitigating effect of hexyl treatment on lung pathology in this model, and (3) no evidence for toxicity of hexyl at the dose studied.

## 1. Introduction

Radiation-induced pulmonary injury causes substantial morbidity and mortality in both accidentally and therapeutically exposed individuals. Single-dose exposures of greater than 9 Gy in human patients induce a subacute inflammatory response (radiation pneumonitis) approximately 2–3 months after exposure, and a more chronic, irreversible syndrome of pulmonary interstitial fibrosis occurring months to years after exposure [1,2]. Among patients receiving fractionated radiotherapy for malignancies of the thorax, the likelihood of lung injury is increased by irradiation of the inferior thorax and by a higher mean dose for normal lungs [3]. The standard of treatment for radiation pneumonitis is high-dose corticosteroids [4]. There is currently no treatment for radiation-induced fibrosis. As mortality due to the hematopoietic syndrome has diminished due to bone marrow transplant and cytokine therapy, pulmonary injury has emerged as an increasingly relevant syndrome. Some survivors of the Chernobyl accident were successfully treated for acute hematopoietic syndrome, only to succumb to pulmonary disease [5]. 

**Figure 1:**
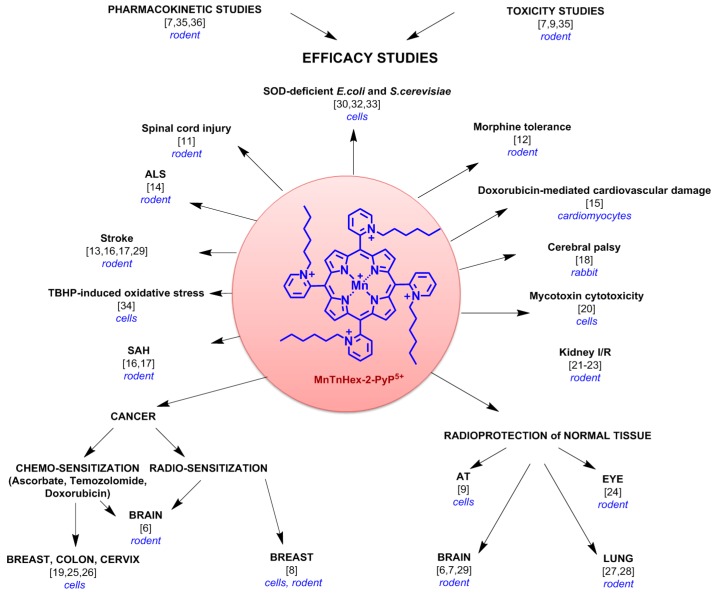
Structure and therapeutic effects of manganese (III) *meso*-tetrakis (*N*-hexylpyridinium-2-yl) porphyrin, MnTnHex-2-PyP^5+^ (hexyl). The therapeutic efficacy of hexyl was observed at the level of normal tissue injury and cancer. Hexyl protected normal tissue from oxidative stress injury, including radiation, at extremely low levels of 0.05 mg/kg. Yet, it did not protect cancer; moreover, it sensitized cancer towards radiation and chemotherapy. The therapeutic effects are detailed in text and summarized in references [6,7,8,9,10,11,12,13,14,15,16,17,18,19,20,21,22,23,24,25,26,27,28,29,30,31,32,33,34,37,40,42]. SAH—Subarachnoid Hemorrhage; I/R—Ischemia/Reperfusion; AT—Ataxia Telangiectasia; ALS—Amyotrophic Laterial Sclerosis; TSHP—*t*-butylhydroperoxide.

Based on impressive therapeutic efficacy in different cellular and animal models of oxidative stress injury [6,7,8,9,10,11,12,13,14,15,16,17,18,19,20,21,22,23,24,25,26,27,28,29,30,31,32,33,34], high lipophilicity [6,29,35], high mitochondrial accumulation [6,36] and good safety/toxicity profile [6,7,9,35], the potent superoxide dismutase (SOD) mimetic and redox-active drug, manganese (III) *meso-*tetrakis (*N*-hexylpyridinium-2-yl) porphyrin, MnTnHex-2-PyP^5+^ (hexyl), was chosen as a mitigator in this nonhuman primate (NHP) model of pulmonary radiation injury (Figure 1). In a rat model of lung radiation injury, we have shown previously that MnTnHex-2-PyP^5+^ was superior to its ethyl analogs, Mn(III) *meso*-tetrakis(*N*-ethylpyridinium-2-yl)porphyrin, MnTE-2-PyP^5+^ (compound name: AEOL10113, BMX-010), as it mitigated radiation-induced lung injury when given at a 120-fold lower dose of 0.05 mg/kg/day for 2 weeks starting at 2 h after irradiation [27,28]. The reason for such dramatic difference in efficacy lies in the more than four orders of magnitude higher lipophilicity of hexyl relative to MnTE-2-PyP^5+^ [6,35,37,38], which, in turn, controls its higher biodistribution and mitochondrial accumulation; both analogs have very similar SOD-like activity [6,37]. The lipophilicity of hexyl is the highest within the class of water-soluble cationic *N*-alkylpyridylporphyrins presently explored [6,29,38]. Therefore, MnTnHex-2-PyP^5+^ distributes in all tissues thus far studied to higher levels than MnTE-2-PyP^5+^ [7,35,39], and is thus up to 120-fold more efficacious in different animal and cellular models, such as SOD-deficient *Escherichia coli* and *Saccharomyces cerevisiae*, yeast, pain, cerebral palsy, stroke, subarachnoid hemorrhage, ischemia/reperfusion models and ataxia telangiectasia (Figure 1) [6,7,8,9,10,11,12,13,14,15,16,17,18,19,20,21,22,23,24,25,26,27,28,29,30,31,32,33,34,37,40]. Such high efficacy allows for ~16-fold larger therapeutic window of MnTnHex-2-PyP^5+^ than of MnTE-2-PyP^5+^ (see details further below in Introduction) [6,9,35]. In addition to normal tissue radioprotection, the radiosensitization of brain, melanoma and breast tumors with hexyl was also demonstrated in mouse models [6,8]. Hexyl and its analogs accumulate in mitochondria [6,15,37] and there mimic the superoxide dismutase isoform, MnSOD [6,15,18,23,40,41]. High lipophilicity allows hexyl to accumulate in both heart and brain mitochondria; while MnTE-2-PyP^5+^ was found in brain [7,35,36,39] it was not found in brain mitochondria [6,29,36]. Hexyl readily crosses the blood–brain barrier [7,29,36] and is the only cationic Mn porphyrin that suppresses infarct volume in a rodent middle cerebral artery occlusion stroke model when given subcutaneously [13,16,29]. Such studies point to the critical role of bioavailability in the therapeutic effects of Mn porphyrins. 

While MnTnHex-2-PyP^5+^ is a potent SOD mimetic, most recent studies point to its role and role of its porphyrin analogs (MnTE-2-PyP^5+^ and MnTnBuOE-2-PyP^5+^) in the modification of the activity of transcription factors including nuclear factor kappa B (NF-κB) and nuclear factor E2-related factor 2 (Nrf2) [22,43,44,45,46] and different mitogen-activated protein kinases, such as extracellular signal-regulated kinase (ERK), c-Jun N-terminal kinase (JNK), protein kinase B (AKT) and p38 mitogen-activated protein kinase (p38-MAPK) [8,45,47]. Several studies provided evidence that this and other potent cationic porphyrin-based SOD mimics (such as MnTE-2-PyP^5+^ and MnTnBuOE-2-PyP^5+^) modify protein cysteines of transcription factors [44,45,46], MAPK, and protein phosphatase 2A through their S-glutathionylation, thereby modifying their activities [45,47]. We have provided the evidence and the mechanistic basis that the SOD-like potency of Mn porphyrins is proportional to their ability to *S*-glutathionylate cysteines of proteins [6,29,47]. 

MnTnHex-2-PyP^5+^ exhibited therapeutic potential as a broadly applicable mitigator of oxidative stress injury and radioprotector of normal tissue, while demonstrating anticancer effects (Figure 1 and references therein). Its high lipophilicity allows it to cross the blood–brain barrier and accumulate in mitochondria [6,7,36]. Its impact on the radioprotection of lungs in a rat model was thoroughly explored [27,28]. MnTnHex-2-PyP^5+^ inhibited the hypoxia inducible factor 1-alpha (HIF-1α) pathway and its gene, vascular endothelial growth factor (VEGF), reduced oxidative stress and suppressed levels of transforming growth factor beta-1 (TGF-β1) [27,28]. Such data suggests that hexyl has the potential to suppress fibrotic processes. A prolonged dosing regimen is required as fibrosis is a late lung injury event. The inhibition of HIF-1α might have occurred via inhibition of NF-κB, which controls HIF-1α and is a major protein affected by Mn porphyrins (see above). In addition to lessening the normal tissue injuries, MnTnHex-2-PyP^5+^ is also an anticancer agent. It suppressed tumor growth, acting as a tumor radio- and chemo-sensitizer in a patient-derived glioblastoma multiforme subcutaneous xenograft mouse model [6]. MnTnHex-2-PyP^5+^ also acted as a tumor radiosensitizer in 4T1 mouse breast and B16 mouse melanoma sc flank models [8]. 

In rodent safety/toxicity studies, the toxicity dose (TD_50_), of MnTnHex-2-PyP^5+^ was determined to be 12.5 mg/kg [9]. That dose causes shivers and hypotonia which is, in part, due to the blood pressure drop. Given its efficacy at a very low 0.05 mg/kg dose, MnTnHex-2-PyP^5+^ (12.5/0.05 = 250) has a 16.3-fold (250/15.3) wider therapeutic window than clinically tested MnTE-2-PyP^5+^. The TD_50_ of 91.5 mg/kg and efficacy dose of 6 mg/kg (91.5/6 = 15.3) were determined for MnTE-2-PyP^5+^ in a parallel study [9]. In a mouse study, at the end of twice sc daily injections of 0.5–2.5 mg/kg for 4 weeks, we have seen the acute degeneration of hippocampal neurons, mild subcutaneous inflammation, degeneration and regeneration of subcutaneous muscles, and pigment accumulation in Kupffer cells of mice [7,35]. However, all mice recovered with no overt pathological changes four weeks after the injections of MnTnHex-2-PyP^5+^ ceased [7,35]. 

The purpose of this study was to evaluate the efficacy and safety of MnTnHex-2-PyP^5+^ in a novel nonhuman primate model of radiation induced lung injury. MnTnHex-2-PyP^5+^ accumulates in lungs to a high level [35], and the mitigation of rat pulmonary radiation injury at 0.05 mg/kg/day justified the dosing schedule used in this study. 

The high clinical prospects of the whole class of cationic Mn porphyrins is best evidenced in several ongoing clinical trials on two analogs of MnTnHex-2-PyP^5+^. Mn(III) *meso*-tetrakis(*N*-n-butoxyethylpyridinium-2-yl)porphyrin, MnTnBuOE-2-PyP^5+^ (BMX-001), is presently being tested as a radioprotector of normal tissue with glioma, head and neck, and anal cancer patients (National Institutes of Health Clinical Trial numbers NCT02655601, NCT02990468 and NCT03386500). MnTE-2-PyP^5+^ (AEOL10113) is also in clinical development. Aeolus Pharmaceuticals is initiating Phase I trials on the di-imidazolyl analog, MnTDE-2-ImP^5+^ (AEOL10150) as a pulmonary radioprotector [48,49]. Such clinical development further justifies the exploration of the therapeutic effects of Mn porphyrins and the studies of their mechanism of action.

The primate model developed for this study was chosen because the respiratory system of nonhuman primates (NHP) is anatomically and physiologically more similar to human beings than that of rodents; respiratory branching patterns are more complex in primates [50]. The resident inflammatory cell population also differs, with rodent lungs containing larger numbers of mast cells with the potential to release histamine and other inflammatory mediators. Primates also provide a large animal model, which allows more precise modeling and measurement of organ dosimetry. The relative sensitivity of mice, in particular, to pulmonary irradiation varies widely [51]. While rodent models allow a degree of genetic manipulation not possible in other species, NHP are a critical element of the late-stage translational application of promising interventions, particularly those that have a mechanism of action that may be primate specific. NHP provides the closest approximation in this regard with respect to the Food and Drug Administration’s Animal Rule, for approval of novel agents which cannot be tested in human subjects [52]. They are also sufficiently long-lived and robust, to allow longitudinal assessment of an array of clinical outcomes, resulting in better pathophysiologic characterization of disease progression than is possible in rodent models. 

## 2. Materials and Methods

### 2.1. Animals

Sixteen juvenile, Chinese-origin, male, rhesus macaques, weighing 3.3–5.7 (mean 3.9) kg, were obtained from a commercial vendor (AlphaGenesis, Yemassee, SC, USA). Animals were pre-screened to exclude simian retrovirus infection and tuberculosis, including quarantine under Centers for Disease Control guidelines. Animals were socially housed in pairs. All animal procedures were performed in accordance with the Guide for the Care and Use of Laboratory Animals [53] and followed protocols for avoidance of pain and discomfort and the assurance of environmental enrichment and psychological well-being, approved by the Wake Forest School of Medicine (WFSM) (Office for Protection from Research Risks #A-3391-01) Institutional Animal Care and Use Committee. WFSM is accredited by the Association for the Assessment and Accreditation of Laboratory Animal Care International and operates in compliance with the Animal Welfare Act. 

### 2.2. Irradiation and Drug Treatment

Animals were randomized to treatment group based on body weight. Treatment groups included 10 Gy whole thorax irradiation (WTI)/sham drug treatment (10 Gy, *n* = 5), 10 Gy WTI with hexyl treatment (10 Gy + hexyl 0.05 mg/kg twice daily subcutaneously, *n* = 5), sham irradiation/sham drug treatment (Control, *n* = 3), and sham WTI with hexyl (Hexyl, *n* = 3). Hexyl was synthesized as previously described [54]. Each NHP to be irradiated was anesthetized with ketamine (5–15 mg/kg, subcutaneously) and dexmedetomidine (0.0075–0.015 mg/kg, subcutaneously) and placed supine with arms extended overhead and lightly restrained to prevent motion. A single fraction dose of 10 Gy was delivered, calculated for each beam at the midline (xiphoid process, nominal depth 4.5 cm), using high energy, 6 megavolt (MV) X rays from a clinical linear accelerator (Varian Medical Systems, Palo Alto, CA, USA). This dose was delivered using a pair of isocentric, equally weighted, parallel-opposed anterior and posterior beams with an asymmetric field size of 10 × 12.5 cm^2^ and nominal dose rate of 200 cGy/min. The anterior beam included a 1-cm flexible slab of tissue-equivalent material on the anterior chest to ensure dose build-up to the anterior lung surface, and a 15-degree physical wedge with the thick end oriented to the superior, as a missing tissue compensator to provide better dose homogeneity at the midline of the mediastinum due to the slope of the anterior surface along the sagittal mid-plane. The central axis was placed through the xyphoid, and the irradiation geometry was confirmed for the anterior beam, which was then irradiated (5 Gy). Using isocentric gantry rotation, the opposed posterior field was set, confirmed and then irradiated (5 Gy). Dose calculations were performed for each beam, with the dose specified at the midline, assuming water-equivalency and without inhomogeneity corrections. Dose inhomogeneity in the lungs for 6 MV X rays is estimated to be a maximum of +10%, found along the greatest anterior–posterior lung diameter, compared to the midplane dose of 10 Gy in the mediastinum. The radiation field superior–inferior borders ranged from the thoracic inlet to 4 cm below the xyphoid process. The radiation field left–right borders flashed beyond the external skin surface of the thorax. Thus, the irradiated region included both lungs (superior to 4 cm below the xyphoid) and the contents of the thorax, including the heart and mediastinum and adjacent portions of the trachea, esophagus, and superior aspects of the liver and stomach.

Animals were given hexyl subcutaneously, twice daily, at a dose of 0.05 mg/kg/dose (0.1 mg/kg/day), for two months after irradiation. Treatment was initated two hours after irradiation by subcutaneous injection. After the initial loading dose, steady state concentrations were administered by subcutaneous implantation of an osmotic pump (Alzet, Durect Corporation, Cupertino, CA, USA). For this procedure, animals were sedated with ketamine, anesthetized with isoflurane, and pumps were placed in the subcutaneous tissues of the upper back using the aseptic surgical technique. Animals not treated with hexyl were given a sham implant or injection containing normal saline. Due to concerns regarding implant surgeries as a complicating factor for interpreting white blood cell counts, at week 6, all pumps were removed and hexyl was again given by twice-daily subcutaneous injection, on alternating sides in the subcutaneous tissue of the torso (to approximate the same physiological site as the injection pump), until the end of the 8-week dosing period. 

### 2.3. Clinical Assessments

Animals were observed daily throughout the study. Respiratory rate and effort were evaluated daily. Treatment-induced morbidity was monitored and documented using a modification of the Children’s Clinical Oncology Group toxicity criteria, customized for nonhuman primates [55]. Animals were lightly sedated with ketamine, weekly, for physical examination and blood collection for complete blood counts and serum chemistries (albumin, bicarbonate, globulin, total protein, electrolytes, triglycerides, bilirubin, hepatic and pancreatic enzymes, blood urea nitrogen, creatinine, creatine kinase, glucose, triglycerides, and cholesterol). Respiratory rates and oxygenation by pulse oximetry (Cardell Veterinary Monitor 9403, Midmark Corp, Versailles, OH, USA) were documented weekly. Normal respiratory rate was considered to be 50 breaths per minute (bpm). The most relevant thresholds for treatment or humane euthanasia were as follows: animals with respiratory rate (RR) >80 bpm were treated with corticosteroids (prednisone, 1 mg/kg/day, tapered). Animals with elevated neutrophil counts were treated with broad spectrum antibiotics (enrofloxacin 5 mg/kg and penicillin, 20,000–60,000 U/kg/day, for 5–8 days). Treatment decisions were made by a veterinary clinician (GD) blinded to treatment group. Animals with RR >100 bpm were euthanized. Veterinary consultation was provided for all animals showing signs of illness, and supportive fluid therapy, corticosteroids, analgesics, antibiotics, and other symptomatic care were given, as needed, based on clinical signs and clinical pathology findings.

### 2.4. Computed Tomography Imaging

All animals were evaluated by computed tomography (CT) imaging prior to irradiation, two months after irradiation, and 4 months after irradiation. Images were acquired on a 16-slice Siemens Biography computed tomography unit, without contrast, at a slice thickness of 0.5 cm, using a pediatric protocol. For this procedure, animals were sedated with ketamine and medetomidine, an endotracheal tube was placed, and anesthesia was induced and maintained with isoflurane. The lungs were fully inflated by manual pressure to the rebreathing bag for a brief breath-hold (approximately 10–20 s) during the scan to avoid motion artifacts. Regions of increased density in the lung were measured from image segmentations performed using Mimics v12.11 software (Materialise, Ann Arbor, MI, USA). A semiautomated method was used to isolate the volumes of air, normal lung tissue, and abnormally dense tissue within the lungs. Automatic thresholding, region growing, morphology, and Boolean operations were utilized, along with manual editing, to create masks representing each volume. Segmentations were performed by a single observer, blinded to treatment group. Three-dimensional reconstruction and volumetric calculations were used to determine the proportion of the lung occupied by air, normal lung tissue, and abnormally dense tissue [56,57,58,59]. 

### 2.5. Pathology

After 19 weeks of observation, all animals were humanely euthanized by intravenous pentobarbital overdose after sedation by intramuscular injection of ketamine. A complete necropsy examination was done for each animal, including gross and histologic examination of all major organ systems, organ weight measurements, and documentation of all abnormalities. Fixed and frozen tissue samples from right and left cranial, middle, and caudal lung lobes were collected. For tissues spanning the border of the radiation field (e.g., trachea, esophagus, skin, and spinal cord), tissues from within and outside the field were examined. Tissues for histology were fixed in 10% neutral buffered formalin, trimmed and embedded in paraffin, sectioned at a 5 micron thickness, and stained with hematoxylin and eosin. Lung sections were also stained with Masson’s Trichrome stain for the identification of fibrosis, and by immunohistochemistry using the human alveolar macrophage marker, HAM56 (Dako, Carpinteria, CA, USA). Histologic examination of the lungs was done by an experienced veterinary pathologist (DLP), blinded to treatment group, and the distribution and amount of qualitative changes were estimated. Additionally, the histologic percentage of the lung parenchyma consisting of macrophages (% positive for the HAM56 macrophage marker by immunohistochemistry), and fibrosis (% section area positive using Masson’s Trichrome stain) were measured on six sections from each animal (one per major lung lobe). Entire sections were measured using computer assisted color image analysis (Image Pro Plus, v. 5.1, Media Cybernetics, Silver Spring, MD, USA). 

### 2.6. Statistical Considerations.

The study was analyzed as a 2 × 2 factorial design with irradiation and hexyl treatment as the independent variables. Dependent variables included respiratory rate, neutrophil count, body weight, lung weight, presence or absence of CT abnormalities, percentage of abnormally dense lung in CT images, and percentage area of macrophages and fibrosis in histologic sections. Parametric methods were used for analysis. For longitudinal data, change from baseline was analyzed, and a multivariate model with repeated measures was used. Fisher’s exact test was used to test differences between groups in days to treatment. The R statistical environment [R] was used for conducting these analyses.

## 3. Results

### 3.1. Clinical Observations

**Figure 2:**
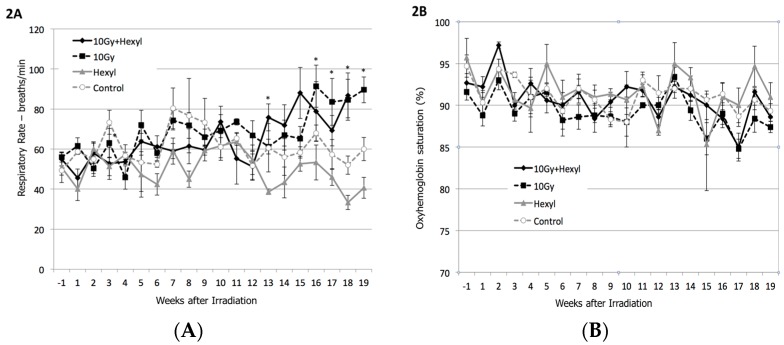
Clinical assessments of pulmonary injury. (**A**) Respiratory rates; irradiated groups differ from non-irradiated animals (*p* = 0.008); * indicates individual time point differences at *p* < 0.05 (*t*-test). (**B**) Oxyhemoglobin saturation. Groups do not differ. Error bars are SEM (standard error of the mean).

During the course of the study, mean respiratory rates increased gradually in irradiated animals, reaching statistical significance by 4 months post-irradiation (*p* < 0.009, Welch two-sample *t*-test) (Figure 2A). Mean oxygenated hemoglobin measurements by pulse oximetry did not change over the course of the study (Figure 2B), suggesting successful compensation by an increased respiratory rate. Irradiated animals were more often treated with antibiotics or corticosteroids, and were treated sooner, more often, and for more total days than non-irradiated animals. The mean number of days to corticosteroid treatment was significantly prolonged for 10 Gy + hexyl-treated animals compared to 10 Gy alone (Table 1). One animal at 4 months crossed the predetermined respiratory rate threshold of 100 bpm, and euthanasia was elected slightly in advance of the remaining animals. No overt respiratory distress or cyanosis was seen in any animal. There were no significant treatment-related changes in body weight during the study (data not shown).

### 3.2. Clinical Pathology

**Figure 3:**
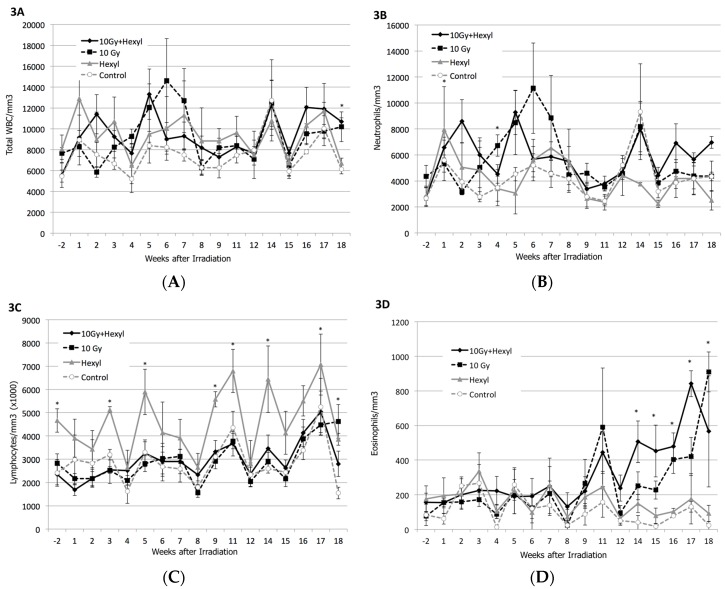
White blood cell changes. (**A**) Overall white blood cell count; (**B**) neutrophil count; (**C**) lymphocyte count; and (**D**) eosinophil count. White blood cell counts (primarily neutrophils) were elevated in irradiated animals 6 weeks after irradiation, coincident with the onset of clinical signs and computed tomography (CT) abnormalities. Lymphocyte counts were highest in non-irradiated hexyl-only animals, and decreased each month after sedation for physical examination. Eosinophil counts were elevated in months 3–5. Error bars are SEM; * = mean differs from control group (*p* < 0.05) (*t*-test).

Elevations in total white blood cell (WBC) count were seen in both groups of irradiated animals, regardless of the presence of hexyl; this change was greater in irradiated animals that did not receive hexyl and reached a peak at about 6 weeks post-irradiation, followed by a decline (Figure 3A). This finding was interpreted as reflecting radiation-induced pneumonitis and consisted primarily of a neutrophilic response (Figure 3B). Lymphocyte counts were higher in the control group at baseline, and this difference persisted throughout the study. Declines in lymphocyte counts were seen in all animals, corresponding to the dates of monthly anesthesia for imaging or osmotic pump implantation (Figure 3C); this was interpreted as a transient stress response. In the final month of the study, eosinophil counts rose markedly in both irradiated groups (Figure 3D). No treatment-related changes were seen in any clinical chemistry parameters (Supplemental Table S1). 

### 3.3. CT Imaging

**Figure 4:**
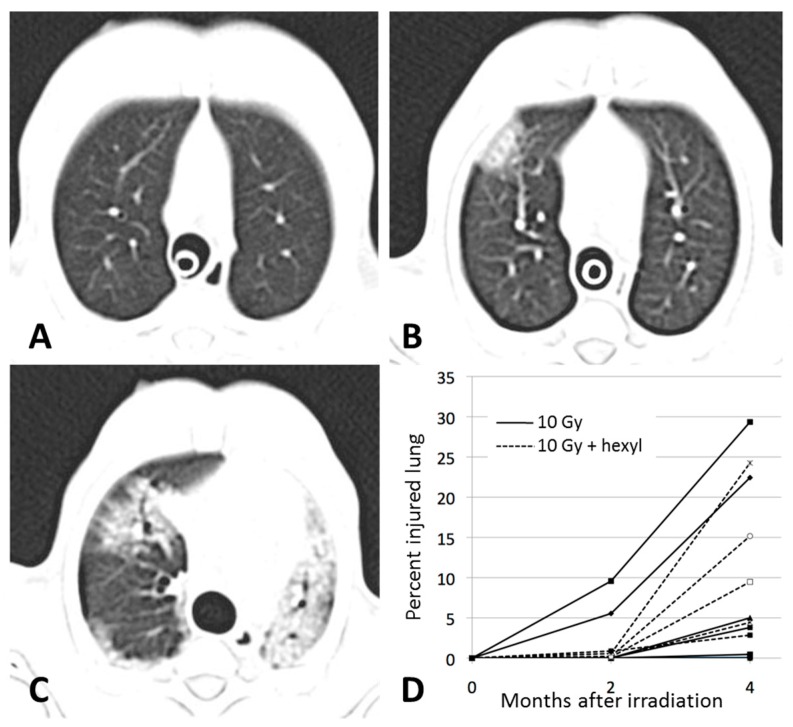
Computed tomography assessments of pulmonary injury. (**A**–**C**) Sequential images from a single animal from the irradiation-only group before irradiation (**A**), 2 months after irradiation (**B**), and 4 months after irradiation (**C**). (**D**) Percentage of injured lung by treatment group over the course of the study. Control and hexyl-alone groups are not shown (all values were <1%). CT abnormalities were seen at 2 and 4 months post-irradiation. The CT density area appears to be lower in hexyl-treated animals, particularly at 2 months, but there is no statistically significant difference.

**Figure 5:**
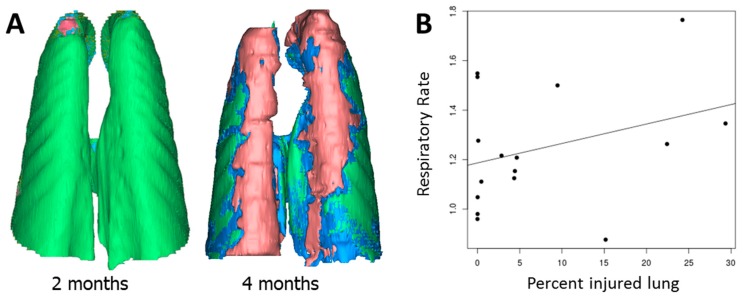
Three-dimensional reconstruction of pulmonary injury in the same animal illustrated in Figure 4 at two and four months after irradiation. The normal lung is green, the partially inflated lung is blue, and the fibrotic/consolidated lung is red (**A**), the dot plot of CT density and respiratory rate at 4 months post-irradiation with linear regression line (*r* = 0.6289) (**B**).

Imaging abnormalities, defined as abnormal radio-opacities exceeding 1% of the lung volume, were seen two months after irradiation in 2/5 animals given irradiation alone. Abnormalities were seen in all irradiated animals at 4 months. These consisted of multifocal, irregular, randomly distributed areas of increased radio-opacity in all lung lobes, which progressed in severity during the course of the study (Figure 4 and Figure 5). The mean volumetric percentage of the lungs occupied by abnormally high density increased; it was minimal at two months and higher at 4 months in irradiated animals treated with hexyl (Figure 4D); this difference was not statistically significant. However, at the 4 month assessment, CT density correlated with elevated respiratory rate (*r* = 0.629, *p* < 0.009) (Figure 5B). 

### 3.4. Pathology Findings

**Figure 6:**
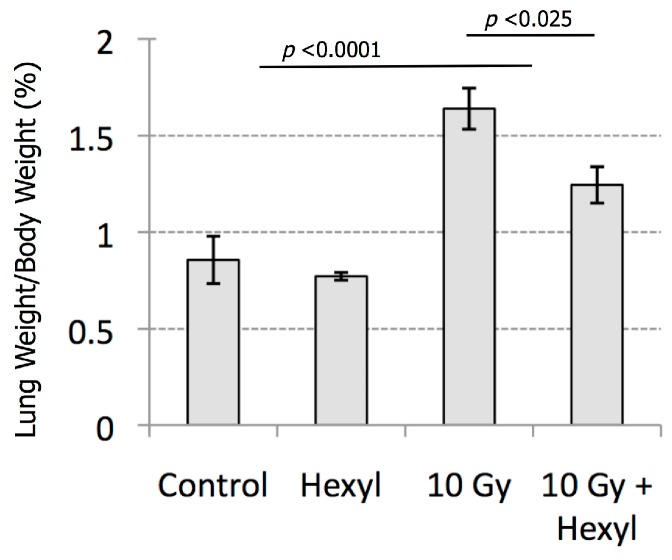
Mean lung weight at necropsy, by treatment group. The adverse main effect of irradiation is significant (*p* < 0.0001), as is the beneficial effect of hexyl treatment (*p* = 0.02418).

Gross abnormalities were limited to animals exposed to radiation, and consisted of increased lung weights, abnormally firm lung consistency on palpation, and multifocal to diffuse, gray to tan discoloration of the pulmonary parenchyma, involving up to approximately 90% of the lung parenchyma. Lung weights in irradiated animals were 77% higher than non-irradiated controls (*p* < 0.0001). These effects were somewhat mitigated by hexyl treatment; irradiated animals treated with hexyl had a 32% lower mean lung weight than those treated with radiation alone (*p* = 0.02; Figure 6). 

**Figure 7:**
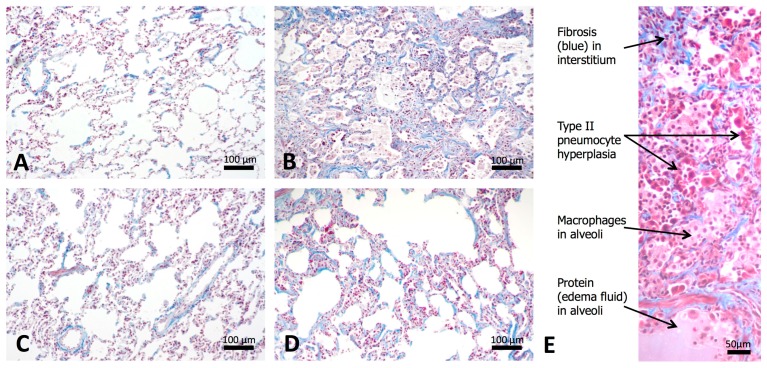
Histologic appearance of lung tissue in (**A**) sham-irradiated, saline-treated controls, (**B**) irradiated animals with no hexyl treatment, (**C**) sham-irradiated animals given hexyl treatment, and (**D**) irradiated animals given hexyl. Trichrome stain. (**E**) Higher-magnification photograph demonstrating specific histopathologic findings. Scale bars: A–D, 100 µm; E, 50 µm.

**Figure 8:**
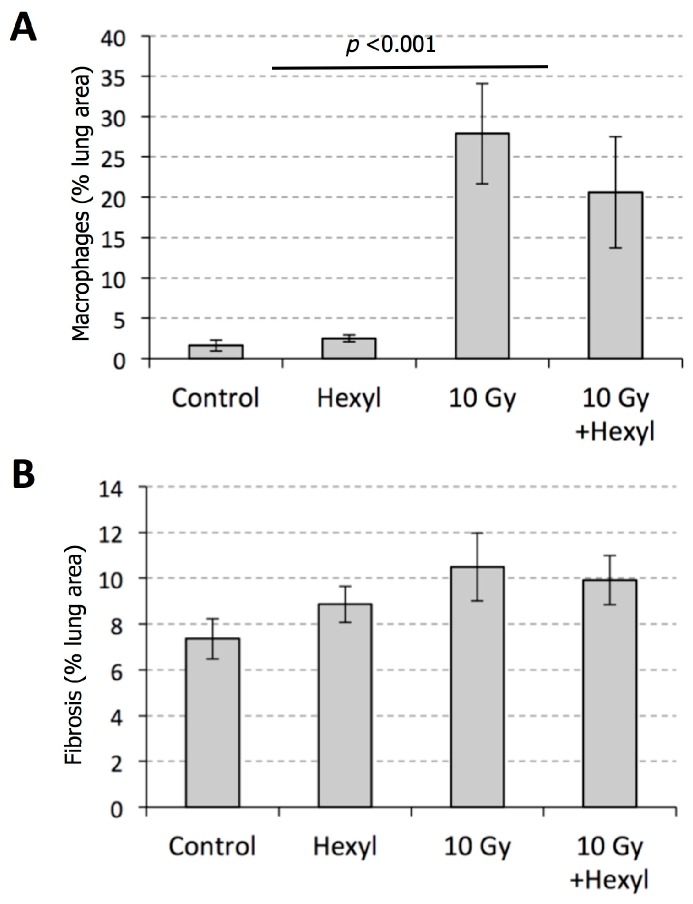
Pulmonary histomorphometry. (**A**) The main effect of radiation was significant (*p* < 0.001) for macrophages expressed as % area of the lung staining for human alveolar macrophage 56 (HAM56) antigen. (**B**) Pulmonary fibrosis was not statistically increased during the course of this experiment.

Histologically, affected regions of the lung in irradiated animals contained four major changes: (1) interstitial and intra-alveolar infiltration of the lung parenchyma by macrophages and other inflammatory cells, (2) accumulation of proteinaceous fluid in alveolar spaces, (3) hyperplasia and hypertrophy of alveolar lining cells, leading to the replacement of the normal thin oxygen exchange layer of type I pneumocytes with a thicker layer of cuboidal Type II cells, and (4) fibrosis of the pulmonary interstitium. These changes and their distribution are illustrated in Figure 7. Notably, the inflammatory, exudative, and hyperplastic changes were diminished in irradiated animals treated with hexyl (Figure 7D), compared to animals given irradiation alone (Figure 7B); this is reflected in the statistically significant difference in lung weights (Figure 6). In contrast, the overall extent of fibrotic changes was not altered by hexyl treatment. Histomorphometric measurements of the proportion of the lung tissue composed of macrophages (HAM56 immunostaining) and fibrous connective tissue (Masson’s Trichrome stain) are shown in Figure 8. While there was a trend towards reduced levels of macrophages by hexyl/10 Gy vs. 10 Gy, statistical significance was not reached. Within lung regions considered fibrotic, the proportion of the tissue occupied by collagen was 14%, compared to 3–5% in non-fibrotic lungs (*p* = 0.0011; data not shown). 

## 4. Discussion

Herein we described the effect of hexyl in a NHP model of radiation-induced lung injury, including an irradiation strategy that that reliably produces pneumonitis and fibrosis within a period of 2–4 months. This model allows longitudinal evaluation of the indicators of disease progression, including clinical evaluations, respiratory rate, oxyhemoglobin saturation, lung density, clinical pathology markers. A clinical and pathologic pattern of disease progression similar to the human disease was documented. 

The major finding in this study was a statistically significant mitigating effect by hexyl treatment on the progression of radiation pneumonitis, as indicated by lung weight, which we attribute to diminished intrapulmonary inflammation and edema. The time course of this effect is interesting in that little or no increase in CT density was seen in hexyl-treated animals at the 2-month time point, whereas most hexyl-treated animals showed some pneumonitis and fibrosis at the final 4-month CT scan, and at 5 months at necropsy. Since treatment was discontinued at 2 months, it is possible that progressive pulmonary disease was, for that time, arrested by hexyl treatment, followed by progressive pneumonitis and fibrosis, beginning after treatment ended. Hexyl cleared from the mouse lungs within 10 days; the t_1/2_ of the elimination of hexyl from the lungs after intraperitoneal injection was 58 h [35]. The mouse and monkey pharmacokinetics are similar (unpublished). We may thus safely assume that hexyl was absent in the lungs post 2 months of injections which would have, therefore, precluded the continuation of its radioprotective effects (observed during 2 months of dosing) and impact on fibrosis. Recently Oberley-Deegan’s team reported the effect of an analogous compound, MnTE-2-PyP^5+^, on the inhibition of a radiation-induced activation of mouse primary prostate fibroblasts via the TGF-β pathway [60]. TGF-β pathway was affected by hexyl and its analog, ethyl, in a mouse/rodent study (see Section 1. Introduction) [27,28]. Insight into the changes in the redox environments of lungs over 4 months of study is essential, as it would allow us to understand events at the molecular level, i.e., discuss the effects of hexyl on the redox-sensitive proteins, low molecular weight antioxidants (such as glutathione and ascorbate) and enzymes. Alternatively, hexyl treatment may have simply shifted the curve for progressive pneumonitis to the right, producing a delay but not an absolute prevention of the disease. 

Due to slow clearance from all tissues [35], less frequent weekly dosing (post tissue loading) maintained throughout the study may prolong the beneficial effects obtained at 2 months post-radiation. Such dosing has been now adopted for the clinical trials on cancer patients for radioprotection of normal tissue with a similarly lipophilic and SOD-active, MnTnBuOE-2-PyP^5+^ [29,33,61,62]. Further work, with longer treatment, less frequent dosing and follow-up will be required to understand how to improve the mitigation of pulmonary injury.

Our findings in untreated NHP are consistent with those of Garofalo et al. [63], namely in regard to progressive pneumonitis and pulmonary fibrosis, affecting all irradiated animals by four months after radiation exposure at 10 Gy. This group showed efficacy of high dose dexamethasone (1 mg/kg/day, tapered) in reducing respiratory rate, which we did not see in our study. This could be related to our choice of corticosteroid (prednisone), which has a shorter duration of activity than dexamethasone. The mean time to corticosteroid therapy was much longer in their work (116 days) relative to our median days to treatment (51 days), despite the use of a similar respiratory rate trigger of 80 breaths per minute; this may reflect the age difference in animals. Our juvenile animals had a baseline respiratory rate of around 50 breaths per minute, whereas the older animals had a lower baseline rate of around 35 breaths per minute [64]. Age could also play a role in radiation sensitivity generally; adolescent rats develop pneumonitis more rapidly than adult animals [65].

The lack of demonstrable benefit for pulmonary fibrosis is disappointing, and may indicate that an additional divergent pathogenetic pathway is involved in fibrosis. Statistical power may have been hampered by high individual variation in responses, the multifocal nature of the fibrosis, and the limited number of animals. We have shown that ongoing oxidative damage and inflammation are important in the pathogenesis of progressive pulmonary disease in rodents, and have also documented involvement of the TGF-beta pathway in radiation-induced pulmonary fibrosis in the rat model [27]. Gene expression studies of the response of primate lungs to ozone-induced oxidative injury also show not only an inflammatory response, with elevations of interleukins 6, 8 and 10, but also a matrix remodeling response, including 3- to 5-fold elevations of matrix metalloproteinase I, metallothionein, and tenascin [66]. Involvement of TGF beta and matrix remodeling pathways may provide additional preventive or therapeutic targets, in addition to oxidative damage pathways. The observation of climbing eosinophil counts in irradiated groups is another interesting finding; eosinophilia was not a prominent feature of the tissue response in the lung, but further studies of lesion progression, for example, focused on hypersensitivity responses or mast cells would be helpful. 

Importantly, in this study, we saw no evidence of toxicity of the hexyl compound, at a therapeutic dose, in a NHP model more closely related to human beings than the rodents studied previously. This drug has also shown efficacy at very low doses in numerous animal models, such as the rat renal ischemia model, the rabbit cerebral palsy model, chronic morphine tolerance, stroke, subarachnoid hemorrhage and tumor radiosensitization [6,7,8,9,10,11,12,13,14,15,16,17,18,19,20,21,22,23,24,25,26,27,28,29]. The efficacy of MnTnHex-2-PyP^5+^ at a low dose (1/120th of the dose used with a hydrophilic MnTE-2-PyP^5+^ analog) is due to its highest lipophilicity within the class of water-soluble cationic *N*-alkylpyridylporphyrins [6,29]. Moreover, due to its lipophilic character and pentacationic charge, this compound crosses the blood–brain barrier, localizes in the hippocampus and other brain parts and accumulates in mitochondria (both brain and heart mitochondria) relative to the cytosol [35,36,40].

Future studies need to address the pharmacokinetics of Mn porphyrin in NHP, explore the impact of the magnitude of the dose and frequency and the duration of dosing and compare MnTnHex-2-PyP^5+^ to another similarly lipophilic and potent SOD-mimic, MnTnBuOE-2-PyP^5+^.

## 5. Conclusions

Data reviewed herein show that lipophilic superoxide dismutase mimetics are effective mitigators of a range of disease processes that involve oxidative injury. New data presented here demonstrate the development of a NHP model of radiation-induced lung injury in a large animal model with high genetic and phenotypic similarity to human beings. Findings indicate potential for mitigation of radiation-induced lung injury by MnTnHex-2-PyP^5+^. The beneficial effect on lung weight but lack of clear effect on anticipated histologic markers suggests that inflammation and fibrosis may not represent the entire picture, and that further mechanistic studies are needed.

## Figures and Tables

**Table 1:** Patterns of antibiotic and corticosteroid treatment; median and range.

Group	Control	Hexyl	10 Gy	10 Gy + Hexyl	Significance *
Number of animals given antibiotics	2/3	2/3	4/5	5/5	nsd
Days to first antibiotic treatment	26 (26–26)	45 (41–48)	31 (26–47)	36 (26–77)	nsd
Number of antibiotic treatments	2 (0–3)	1 (0–1)	3 (0–5)	2 (1–4)	nsd
Total days of antibiotic treatment	11 (0–17)	5 (0–5)	18 (0–31)	12 (5–27)	nsd
Number of animals given prednisone	1/3	0/3	5/5	5/5	nsd
Days to first prednisone treatment	47 (47–47)	NA	51 (26–77)	90 (51–103)	*p* < 0.05
Number of prednisone treatments	1 (NA)	0	2 (1–3)	1 (1–4)	nsd
Total days of prednisone treatment	9 (NA)	0	10 (5–14)	7 (4–21)	nsd

* Fisher’s exact test; nsd = no significant difference; NA: not applicable.

## Supplementary Materials

The following are available online at http://www.mdpi.com/2076-3921/7/3/40/s1.
