# Supplementary material for: Post-Irradiation Treatment with a Superoxide Dismutase Mimic, MnTnHex-2-PyP5+, Mitigates Radiation Injury in the Lungs of Non-Human Primates after Whole-Thorax Exposure to Ionizing Radiation
Source: Antioxidants (Basel). 2018 Mar 7;7(3):40. doi: 10.3390/antiox7030040 (PMC5874526; doi:10.3390/antiox7030040)
Supplement: Supplementary file 1 [file antioxidants-07-00040-s001.pdf]

Post-Irradiation Treatment with a Superoxide Dismutase Mimic, MnTnHex-2-PyP^{5+}, Mitigates Radiation Injury in the Lungs of Non-Human Primates after Whole-Thorax Exposure to Ionizing Radiation

J. Mark Cline [1,*], Greg Dugan [1], J. Daniel Bourland [2], Donna L. Perry [1,3], Joel D. Stitzel [4], Ashley A. Weaver [4], Chen Jiang [5], Artak Tovmasyan [6], Kouros Owzar [5], Ivan Spasojevic [7,8], Ines Batinic-Haberle [6] and Zeljko Vujaskovic [6,9]

[1] Department of Pathology, Section on Comparative Medicine, Wake Forest University School of Medicine, Bowman Gray Center, 475 Vine St, Winston-Salem, NC 27157-1040, USA; gdugan@wakehealth.edu (G.D.); perrydl@niaid.nih.gov (D.L.P.)

[2] Department of Radiation Oncology, Wake Forest University School of Medicine, Bowman Gray Center, 475 Vine St, Winston-Salem, NC 27157-1040, USA; bourland@wakehealth.edu

[3] Integrated Research Facility, Division of Clinical Research, National Institute of Allergy and Infectious Disease, National Institutes of Health, Frederick, MD 21702, USA

[4] Department of Biomedical Engineering, Wake Forest University School of Medicine, Bowman Gray Center, 475 Vine St, Winston-Salem, NC 27157-1040, USA; jstitzel@wakehealth.edu (J.D.S.); asweaver@wakehealth.edu (A.A.W.)

[5] Department of Biostatistics and Bioinformatics, Duke University Medical Center, Durham, NC 27708, USA; chen.jiang@duke.edu (C.J.); kouros.owzar@duke.edu (K.O.)

[6] Department of Radiation Oncology, Duke University Medical Center, Durham, NC 27708, USA; artak.tovmasyan@duke.edu (A.T.); ibatinic@duke.edu (I.B.-H.); zvujaskovic@som.umaryland.edu (Z.V.)

[7] Department of Medicine Duke University Medical Center, Durham, NC 27708, USA; ivan.spasojevic@duke.edu

[8] Duke Cancer Institute, Pharmaceutical Research Shared Resource, PK/PD Core laboratory, Duke University Medical Center, Durham, NC 27708, USA

[9] Department of Radiation Oncology, University of Maryland, Baltimore, MD Zip Code, USA

* **C**orrespondence: jmcline@wakehealth.edu; Tel.: +1+336+716+1564; Fax: +1-336-716-1515

Supplemental Table S1: Serum chemistry values; mean and standard deviation are presented. 17 measurements were made across the study; baseline and 4-month time points (End) are shown.

Serum Analyte	Control Baseline (*n* = 3)	Hexyl Baseline (*n* = 3)	10 Gy Baseline (*n* = 5)	10 Gy + Hexyl Baseline (*n* = 5)	Control End (*n* = 3)	Hexyl End (*n* = 3)	10 Gy End (*n* = 5)	10 Gy + Hexyl End (*n* = 5)	Significant Group Effect (Tukey HSD)
**Albumin mg/dL**	4.5 +/– 0.1	4.4+/-0.4	4.4+/-0.1	4.4+/-0.3	4.5+/-0.1	4.3+/-0.2	3.7+/-0.3	3.9+/-0.2	none
**ALP IU/L**	771 +/– 163	543 +/– 136	537 +/– 114	413 +/– 144	821 +/– 212	543 +/– 123	439 +/– 140	429 +/– 80	none
**ALT IU/L**	46 +/– 18	37 +/– 2	38 +/– 9	49 +/– 23	42 +/– 20	39 +/– 13	46 +/– 20	67 +/– 40	none
**Amylase IU/L**	254 +/– 71	280 +/– 59	288 +/– 48	284 +/– 39	332 +/– 57	352 +/– 41	238 +/– 34	256 +/– 85	$p < 0.01$ (10 Gy vs Control and Hexyl)
**AST IU/L**	41 +/– 1	38 +/– 4	39 +/– 6	44 +/– 11	54 +/– 15	34 +/– 6	62 +/– 27	58 +/– 26	none
**BUN mg/dL**	17 +/– 2	22 +/– 3	20 +/– 2	18 +/– 2	21 +/– 1	21 +/– 4	20 +/– 5	19 +/– 4	none
**Calcium mEq/L**	9.9 +/– 0.4	9.4 +/– 0.3	9.2 +/– 0.2	9.3 +/– 0.3	9.1 +/– 0.5	9.3 +/– 0.3	9.4 +/– 0.4	9.4 +/– 0.3	none
**Chloride mEq/L**	107 +/– 2	107 +/– 1	109 +/– 2	109 +/– 4	106 +/– 3	108 +/– 1	104 +/– 2	105 +/– 4	$p < 0.002$ (10 Gy and 10 Gy + Hexyl vs Hexyl)
**CPK IU/L**	370 +/– 158	308 +/– 8	446 +/– 284	924 +/– 873	1124 +/– 1407	245 +/– 139	803 +/– 759	645 +/– 374	none
**Cr mg/dL**	0.6 +/– 0.1	0.5 +/– 0	0.5 +/– 0.1	0.5 +/– 0.1	0.6 +/– 0.1	0.5 +/– 0.1	0.5 +/– 0.1	0.6 +/– 0.1	none
**GGT IU/L**	88 +/– 11	99 +/– 9	79 +/– 7	73 +/– 8	93 +/– 21	99 +/– 6	60 +/– 17	83 +/– 6	$p = 0.011$ (10 Gy vs 10 Gy + Hexyl)
**Globulin mg/dL**	2.2 +/– 0.1	2.1 +/– 0.2	2.1 +/– 0.1	2.3 +/– 0.2	2.3 +/– 0.1	2.3 +/– 0.2	3.1 +/– 0.3	2.9 +/– 0.4	none
**Glucose mg/dL**	75 +/– 29	60 +/– 11	64 +/– 16	63 +/– 11	48 +/– 43	65 +/– 8	58 +/– 36	49 +/– 14	none
**Lipase IU/L**	22 +/– 3	21 +/– 5	22 +/– 12	26 +/– 11	67 +/– 52	25 +/– 12	12 +/– 5	28 +/– 25	none
**Magnesium mEq/L**	1.4 +/– 0.1	1.5 +/– 0.1	1.5 +/– 0.1	1.5 +/– 0.1	1.8 +/– 0.1	1.6 +/– 0.1	1.7 +/– 0.2	1.6 +/– 0.1	$p <0.01$ (10 Gy vs Control and Hexyl)
**Phosphorus mEq/L**	5.3+/-0.6	5.2+/-1.0	6.2+/-1.0	6.0+/-0.8	6.2+/-0.8	5.7+/-0.1	6.1+/-0.9	6.1+/-0.7	none
**Potassium mEq/L**	3.6 +/– 0.3	3.5 +/– 0.3	3.6 +/– 0.3	3.7 +/– 0.4	4.7 +/– 1.5	3.7 +/– 0.3	4.6 +/– 1.1	3.9 +/– 0.5	none
**Sodium mEq/L**	144 +/– 1	144 +/– 1	145 +/– 2	147 +/– 1	147 +/– 4	146 +/– 1	145 +/– 1	145 +/– 3	none
**Total Bilirubin mg/dL**	0.2 +/– 0.1	0.2 +/– 0.1	0.3 +/– 0.1	0.3 +/– 0.1	0.3 +/– 0.1	0.3 +/– 0.1	0.3 +/– 0.1	0.3 +/– 0.1	none
**TSP mg/dL**	6.7 +/– 0.1	6.5 +/– 0.5	6.5 +/– 0.2	6.7 +/– 0.5	6.8 +/– 0	6.6 +/– 0.1	6.8 +/– 0.3	6.8 +/– 0.4	none

ALP, alkaline phosphatase; ALT, alanine aminotransferase; AST, aspartate aminotransferase; BUN, blood urea nitrogen; CPK, creatine phosphokinase; Cr, creatinine; GGT, gamma-glutamyl transferase; TSP, total serum protein.
